# Determinants and Correlates of Occupational Skin Diseases and Workplace Risk Stratification in a Metal Casting Factory: A Cross-Sectional Study From a Rural Coastal Region of Southern India

**DOI:** 10.7759/cureus.111370

**Published:** 2026-06-23

**Authors:** Srikrishna Sulgodu Ramachandra, Shobhit Mohan, Vishal K Singh, Priya Dubey, Gokuldas V Sawant

**Affiliations:** 1 Community Medicine, Narayan Medical College and Hospital, Sasaram, IND; 2 Dermatology, KMC Medical College and Hospital, Maharajganj, IND; 3 Community Medicine, KMC Medical College and Hospital, Maharajganj, IND

**Keywords:** contact dermatitis, cross-sectional study, factory workers, india, metal casting workers, occupational skin diseases, personal protective equipment, workplace risk stratification

## Abstract

Introduction

Workers in metal casting industries are routinely exposed to high temperatures, hazardous chemicals, metal fumes, and dust, placing them at significant risk of occupational skin diseases (OSD). Despite the considerable morbidity and productivity loss associated with OSD, systematic assessment in the Indian metal casting industry remains limited. This cross-sectional study aimed to assess the prevalence, pattern, and determinants of OSD among workers at a private metal casting factory in Goa, India, and to explore the association between personal protective equipment (PPE) use and OSD occurrence.

Materials and methods

A cross-sectional observational study was conducted at a private limited metal casting factory in Kundaim Industrial Estate, Kundaim, Goa, as part of the mandatory annual free health check-up under the Factories Act, 1948. Of the 414 registered employees, 345 (83.3%) were examined on the day of the check-up. A pretested, pre-validated, and structured questionnaire captured sociodemographic details, work experience, and self-reported and observed PPE use. A dermatologist (MBBS, MD - Dermatology) independently examined all participants and, based on a detailed history and clinical examination, classified skin disorders as occupational or non-occupational. Data were analysed using IBM SPSS Statistics for Windows, Version 26 (Released 2019; IBM Corp., Armonk, New York, United States). Chi-square test, Fisher's exact test, and binary logistic regression were applied, with p<0.05 considered as statistically significant.

Results

The study population was predominantly made up of male subjects (87.5%). The most common age group was 31-45 years (36.5%), and the majority belonged to Class II socioeconomic status (SES) (69.3%). Overall, 121 workers (35.1%) were diagnosed with at least one OSD. Contact dermatitis was the most prevalent condition (15.1%), followed by prickly heat (14.5%), tinea (4.6%), burn scars (3.5%), and urticaria (2.6%). OSD prevalence was significantly associated with age (p=0.027), workplace type (p<0.01), years of work experience (p=0.001), and self-reported PPE use (p<0.01). Among workplace categories, core-making workers had the highest OSD burden (65%). On binary logistic regression, workers who reported to use PPE were 50.8 times more likely to be free of OSD compared to non-users (AOR 50.8; 95% CI: 18.2-141.7; p<0.01).

Conclusion

More than one in three metal casting workers had a clinically diagnosed OSD, with contact dermatitis being the most prevalent condition. Among workplace categories, core-making workers bore the highest burden of OSD. Reported PPE use was the single most significant modifiable protective factor, with users several times more likely to be free of OSD. Targeted interventions including consistent PPE promotion, periodic dermatological screening, and workplace hazard reduction are essential to reduce the burden of OSD in the metal casting industry sector.

## Introduction

The metal casting industry in India includes steel plants and foundries, which are a fairly organized sector, and the automobile repair industry, which is largely unorganized [1]. Workers in these industries are frequently exposed to high temperatures, high levels of noise, hazardous chemicals, metal fumes, and dust [2]. These environments lead to significant occupational hazards, including systemic diseases and skin disorders [3]. The scope of this manuscript is limited to occupational skin diseases (OSD) occurring in the metal casting industry. 

Several common skin diseases occur amongst workers in the metal casting industry [4]. Some of these result from the working conditions prevalent in the workplace, some are related to exposure to various materials and chemicals that the workers are exposed to (occupational hazards), and some are related to the living conditions of these workers [5].

Although there is no mortality associated with OSD, they cause significant morbidity, sickness, and absenteeism, and hence loss of productivity to the employer and to the industry [3]. They also cause loss of wages to the employee [6]. Hence, it is important to periodically assess these skin problems in the metal casting industry and ensure that regular medical check-ups, investigations, and treatment are provided to the employees. It is important to provide a proper orientation and training on preventive measures to prevent OSD [4]. This study was done as a part of the regular annual health check-up of all the employees of the concerned metal casting factory. The factory under discussion is a private limited metal casting factory and foundry specializing in grey and ductile iron castings, founded in 1987 and based in state of Goa, India [7].

Objectives

To assess the patterns of OSD among workers in the metal casting industry; to identify the determinants and correlates of OSD among workers in the metal casting industry; to explore association of OSD with the use of personal protective equipment (PPE); to suggest preventive and management measures to tackle OSD among these workers. 

## Materials and methods

Study design

This was a cross-sectional observational study.

Study location

The factory is a private limited factory located in Kundaim Industrial Estate, Kundaim, Goa, which is a rural area. 

Duration of the study

Data collection was undertaken between the 19th and 30th May, 2025.

Study population

All the employees of the concerned factory (414 workers) were provided with the free annual check-up as per the provisions of the Factories Act, 1948 and as part of the benefits given to the employees towards their health insurance and annual health check-up [8]. On the day of examination, 345 (83%) out of 414 were available. This manuscript’s scope is limited to the OSD identified as part of this annual health check-up.

An MBBS doctor initially examined all the patients for general ailments as part of the general physical examination. For specific skin disorders, a dermatologist (MBBS, MD - Dermatology) examined all the employees of the metal casting factory and categorized the skin diseases as occupation-related or otherwise, based on a detailed history and clinical examination. All the employees who had skin diseases were diagnosed and treated. For skin disorders, the diagnosis and decision made by the dermatologist was considered final.

Data collection tools

A pretested and pre-validated, structured, closed-ended questionnaire was prepared and pilot tested. Based on the results, necessary corrections were made in the questionnaire and then it was finalized and used. The dermatologist provided a case sheet for each of the employees that were examined by him and diagnosis was provided.

Regarding PPE use

An initial question on whether a particular factory worker used PPE or not was asked to the individual and the answer was elicited. At a later point, the factory worker was observed at his/her work station, and based on this, PPE use relevant to their particular workstation was documented.

Ethics approval

Ethics approval for this study was obtained from the Institutional Ethics Committee (IEC) of KMC Medical College and Hospital, Maharajganj, UP (approval number: 15/KMC&H/IEC/2025). An approval from the Office of Medical Inspector of Factories was obtained for collecting data from employees of the factory and also for conducting their medical examination. Data were anonymized before analysis.

Data analysis

Data analysis was performed using Excel (Microsoft Corp., Redmond, WA, USA) and IBM SPSS Statistics for Windows, Version 26 (Released 2019; IBM Corp., Armonk, New York, United States). Frequencies and cross tabulations were done to identify any association between OSD and its determinants. Pearson’s chi square test and Fisher’s exact test were done to identify factors associated with OSD; binary logistic regression analysis was done to identify causality. A p-value of <0.05 was considered significant. In the binary logistic regression, the event modelled was freedom from OSD; accordingly, an adjusted odds ratio (AOR) above one indicates a greater likelihood of remaining disease-free (a protective association), and an AOR below one indicates a greater likelihood of OSD.

## Results

The analysis included 345 factory employees who were present on the day of the annual health check-up. The workforce's sociodemographic profile, the study population's occupational characteristics, the pattern and prevalence of clinically diagnosed OSD, the bivariate relationships between OSD and sociodemographic, occupational, and PPE variables, and a multivariable binary logistic regression of the variables independently linked to OSD comprise the five subsections of the findings. Both frequency and percentages were adopted to present categorical variables, Pearson's chi-square test or Fisher's exact test were used to test associations, and variables found to be significant on bivariate analysis were added to a binary logistic regression model, in which a p-value<0.05 was considered statistically significant. 

Sociodemographic indicators

**Figure 1:**
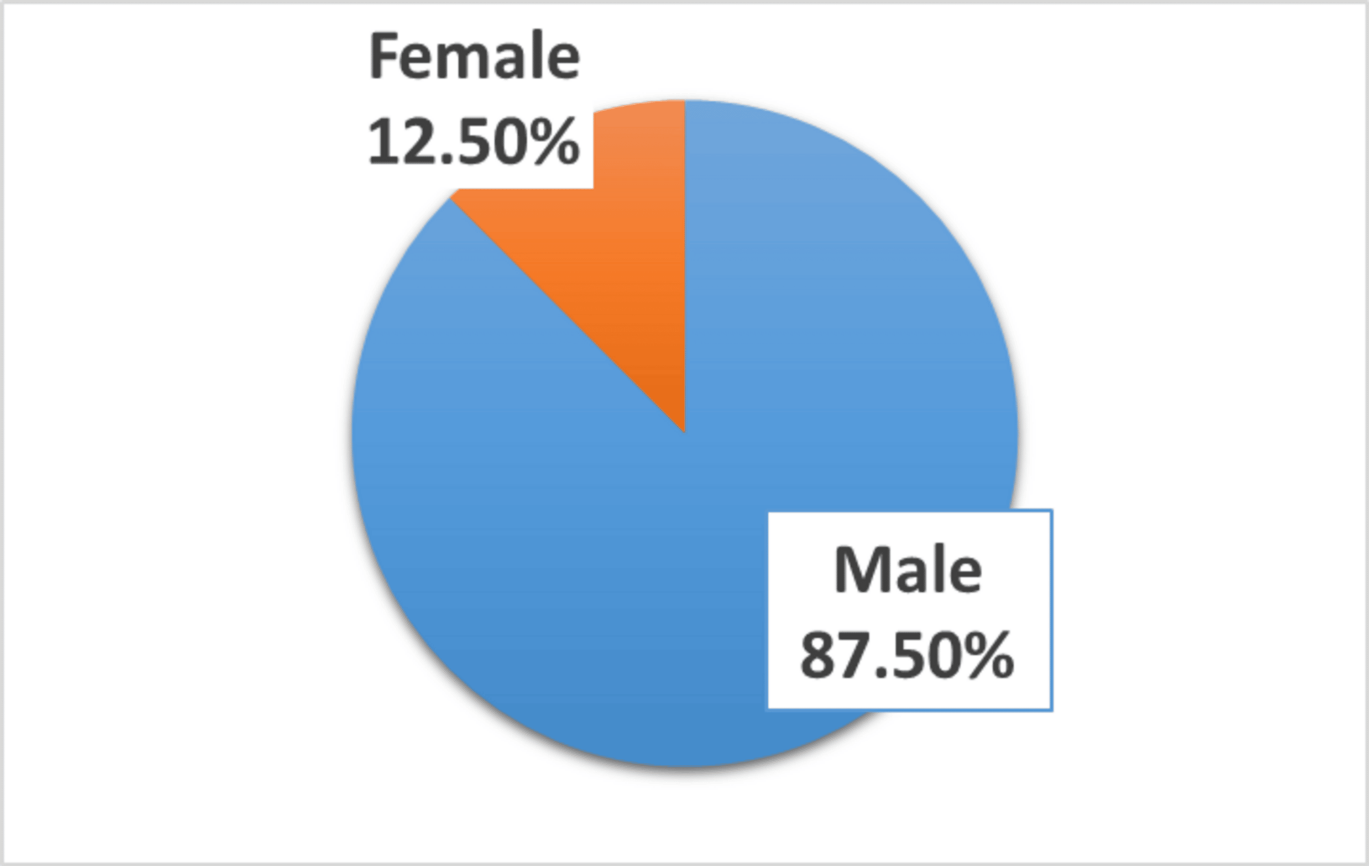
Gender distribution of the industry workers (n=345)

A total of 345 factory workers were included in the analysis. Figure 1 shows the gender distribution of the study population, i.e., the factory workers.

**Figure 2:**
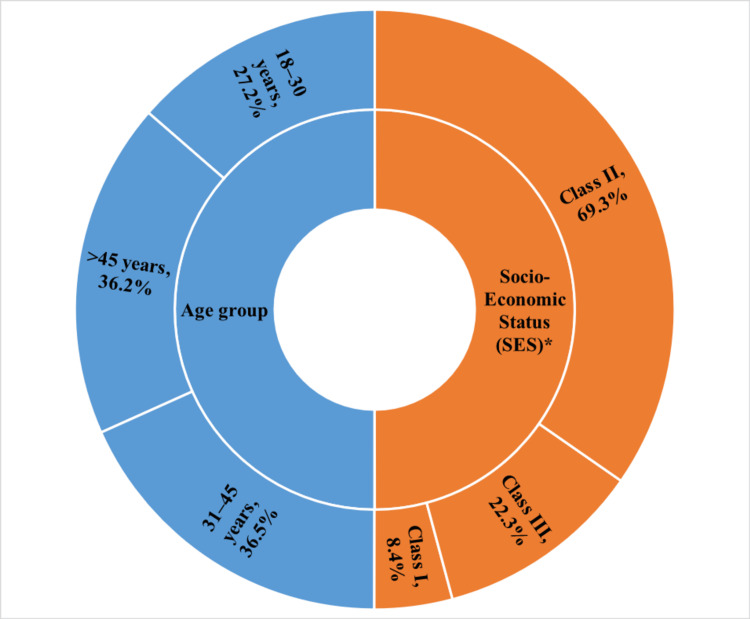
Sociodemographic details of the study participants (n=345) Based on [9].

The study population consisted mainly of male subjects (302 out of 345, 87.5%). Figure 2 shows the distribution of age group and socioeconomic status (SES), according to updated B.G. Prasad scale for industry workers in a rural area [9].

The most frequent age group found in the study was 31 to 45 years (36.5%) and the second-largest age group was more than 45 years. Majority of the participants belonged to Class II SES (n=239; 69.3%).

Occupational characteristics

Table 1 shows the type of workplace, work-related experience in the concerned factory, as well as self-reported and observed use of PPE.

**Table 1:** Workplace, work-related experience, and PPE use (n=345)

Variable	Category	n (%)
Workplace	Housekeeping, canteen & maintenance	67 (19.4%)
	Core making	84 (24.3%)
	Melting & moulding	80 (23.2%)
	Polishing & grinding	114 (33%)
Work experience at the factory	<1 year	68 (19.7%)
	1–5 years	41 (11.9%)
	>5 years	236 (68.4%)
Self-reported personal protective equipment (PPE) use	Yes	283 (82%)
	No	62 (18%)
Flame-resistant coveralls	Yes	149 (43%)
	No	196 (57%)
Heat-resistant gloves	Yes	239 (69%)
	No	106 (31%)
Face mask	Yes	335 (96%)
	No	9 (4%)
Safety boots	Yes	310 (89.9%)
	No	35 (10.1%)
Helmet	Yes	309 (89.5%)
	No	36 (10.5%)
Face shield	Yes	255 (74%)
	No	90 (26%)
Ear muffs	Yes	194 (56%)
	No	151 (44%)

The factory workers according to the place of work were categorised into four categories; namely, housekeeping, canteen & maintenance, core making, melting & moulding, andpolishing & grinding. Among these, majority were found in polishing & grinding (n=114; 33%), followed by core making (n=84; 24.3%), melting and moulding (n=80; 23.2%), and least were found working in housekeeping, canteen, and maintenance category (n=67; 19.4%). Work-related experience was recorded and classified into three categories: <one year, 1-5 years, and >five years. Majority of the factory workers reported that they had more than five years of experience (n=236; 68.4%) followed by less than one year (n=68; 19.7%) and 1-5 years experience (n=41; 11.9%). Of the total participants (n=345), majority (n=283; 82.0%) reported using PPE during work, while others (n=62; 18%) did not use them. The utilization of individual PPE items was further examined. Face masks were worn by majority of workers (n=335; 96%), followed by safety boots (n=310; 89.9%), helmets (n=309; 89.5%), face shields (n=255; 74%), heat-resistant gloves (n=239; 69%), ear muffs (n=194; 56%), and flame-resistant coveralls (n=149; 43%).

Pattern of OSD

**Figure 3:**
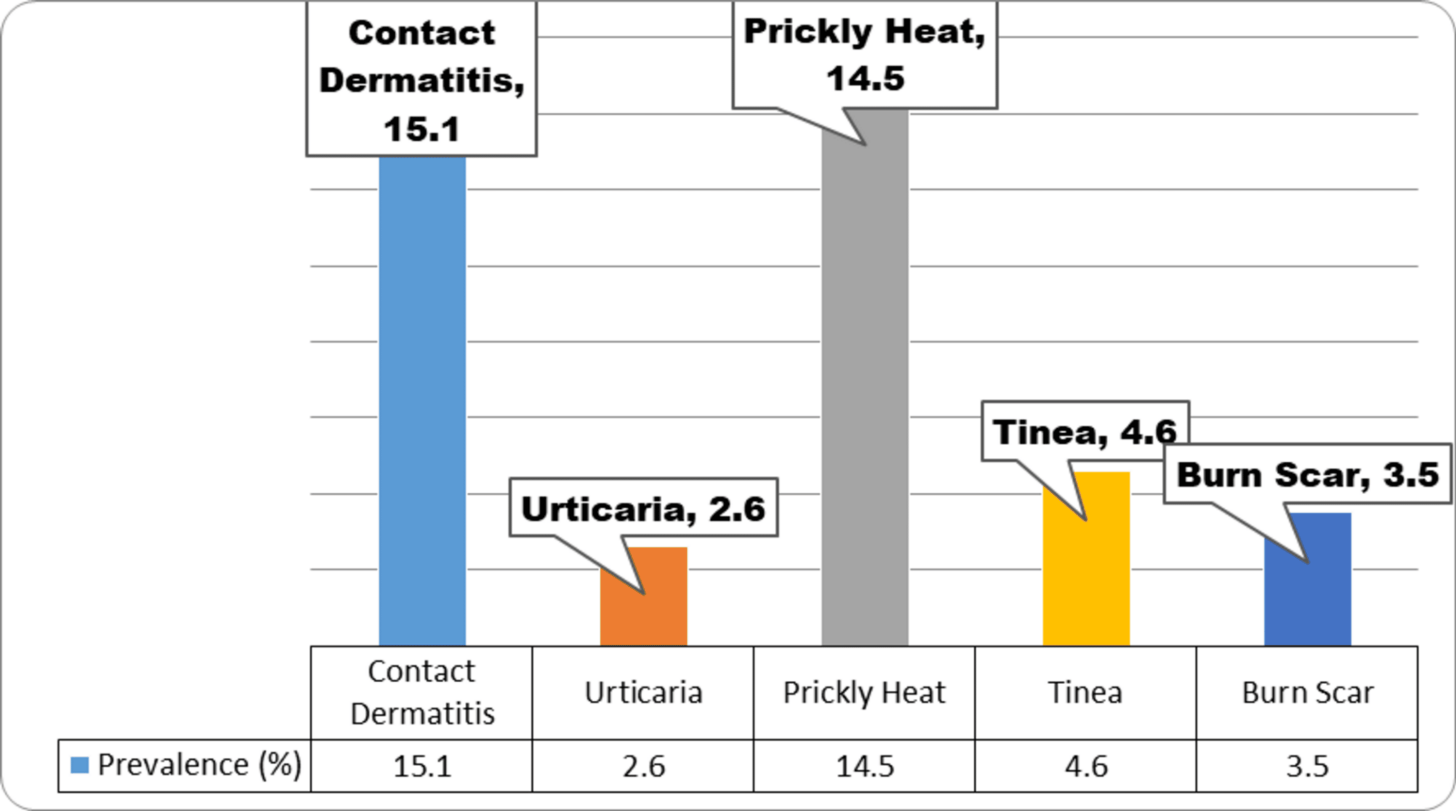
Prevalence of occupational skin diseases (OSD)

Figure 3 presents the prevalence of OSD, clinically diagnosed by a dermatologist, among the factory workers.

Out of the total 345 factory workers that were examined, several were diagnosed with at least one OSD (n=121; 35.1%). Among the skin conditions examined, contact dermatitis was the most prevalent (n=52; 15.1%). Prickly heat was the second most common condition (n=50; 14.5%), followed by tinea (n=16; 4.6%), and burn scars (n=12; 3.5%). Urticaria was the least prevalent condition (n=9; 2.6%).

Correlation of OSD with sociodemographic and other variables

Table 2 presents the cross-tabulation of OSD with various independent variables, with chi-square values and p-values calculated to determine statistical significance.

**Table 2:** Association of sociodemographic and occupational factors with OSD (n=345) OSD: occupational skin diseases. *statistically significant (p<0.05).

Variables		OSD present	OSD absent	Chi-square value	p-value
Gender	Male	101 (33.4%)	201 (66.6%)	2.823	0.09
	Female	20 (46.5%)	23 (53.5%)		
Age	18-30	26 (28%)	68 (72%)	7.25	0.027*
	30-45	40 (32%)	86 (68%)		
	>45	55 (44%)	70 (56%)		
Workplace	Housekeeping, Canteen & Maintenance	22 (33%)	45 (67%)	48.717	<0.01*
	Core Making	55 (65%)	29 (35%)		
	Melting & Moulding	22 (28%)	58 (73%)		
	Polishing & Grinding	22 (19%)	92 (81%)		
Work experience at the concerned factory	<1	11 (16%)	57 (84%)	13.651	0.001*
	1-5	18 (44%)	23 (56%)		
	>5	92 (39%)	144 (61%)		
Self-reported personal protective equipment (PPE) use	Yes	65 (23%)	218 (77%)	101.32	<0.01*
	No	56 (90%)	6 (10%)		

With respect to gender, OSD was present in 101 (33.4%) male workers and 20 (46.5%) female workers. However, the association between gender and OSD was not statistically significant (χ² = 2.823, p=0.09). Regarding age, OSD was observed in 26 (28%) workers in the 18-30 age group, 40 (32%) in the 30-45 age group, and 55 (44%) in those above 45 years. The association between workplace and OSD was statistically significant; OSD was most prevalent among core-making workers (n=55; 65%), followed by housekeeping, canteen and maintenance workers (n=22; 33%), melting and moulding workers (n=22; 28%), and least among polishing and grinding workers (n=22; 19%). Years of work experience also showed a statistically significant association with OSD; workers with one to five years of experience recorded the highest prevalence of OSD (n=18; 44%), followed by those with more than more years (n=92; 39%), while workers with less than one year of experience had the lowest prevalence (n=11; 16%). With respect to self-reported PPE use, majority of the 283 workers who reported using PPE did not have any type of OSD on clinical examination (n=218; 77%), which was found to be statistically significant, while majority out of 62 who reported not using PPE had at least any one type of OSD (n=56; 90%).

Table 3 presents the association between observed specific PPE use and OSD among the factory workers.

**Table 3:** Association between PPE use and occupational skin diseases (OSD) among the factory workers (n=345)

Specific personal protective equipment (PPE) use (observed)		OSD present	OSD absent	Chi-square value (p-value)	Odds ratio (95% CI)
Flame-resistant coverall	Yes	51 (34%)	98 (66%)	0.082 (0.774)	0.937 (0.6-1.4)
	No	70 (36%)	126 (64%)		
Heat-resistant gloves	Yes	80(33.5%)	159 (66.5%)	0.0874 (0.350)	0.8 (0.4-1.2)
	No	41 (39%)	65 (61%)		
Face mask	Yes	117 (35%)	218 (65%)	0.110 (0.740)	0.8 (0.2- 2.9)
	No	4 (40%)	6 (60%)		
Safety boots	Yes	109 (35%)	201 (65%)	0.011 (0.918)	1.04 (0.5- 2.1)
	No	12 (34%)	23 (66%)		
Helmet	Yes	108 (35%)	201 (65%)	0.019 (0.890)	0.95 (0.46-1.95)
	No	13 (36%)	23 (64%)		
Face shield	Yes	88(34.5%)	167 (65.5%)	0.136 (0.712)	0.91 (0.55-1.5)
	No	33(37%)	57 (63.3%)		
Ear muffs	Yes	66 (34%)	128 (66%)	0.215 (0.643)	0.9 (0.57- 1.4)
	No	55(36%)	96 (63.6%)		

Among wearers of flame-resistant coveralls, 34% developed OSD compared to 36% of non-wearers. In the case of heat-resistant gloves, 33.5% wearers developed OSD versus 39% among non-wearers. For face masks, 35% of wearers had OSD compared to 40% among non-wearers. Thirty five percent of safety boots wearers developed OSD versus 34% of non-wearers; similarly, 35% of helmet wearers had OSD compared to 36% of non-wearers. In the case of face shields, 34.5% of wearers developed OSD versus it was 37% among non-wearers. Lastly, 34% of ear muffs wearers had OSD compared to 36% of non-wearers.

The odds of OSD development among flame-resistant coverall wearers versus non-wearers was 0.937 (95% CI: 0.6-1.4). These odds were 0.8 (95% CI: 0.4-1.2), 0.8 (95% CI: 0.2-2.9), 1.04 (95% CI: 0.5-2.1), 0.95 (95% CI: 0.46-1.95), 0.91 (95% CI: 0.55-1.5), and 0.9 (95% CI: 0.57-1.4) for heat resistant gloves, face masks, safety boots, helmets, face shields, and ear muffs, respectively. None of the PPE types showed a statistically significant association with OSD development.

Binary logistic regression

All variables found to be statistically significant, such as age group, workplace, years of experience, and self-reported PPE use, were entered into a logistic regression model, and the results are displayed in Table 4.

**Table 4:** Logistic regression analysis of the factors associated with occupational skin disorders (OSD) among factory workers (n=345) ^Not using the specific PPE as reference category, *statistically significant at p-value<0.05.

Covariate	Odds ratio	95% CI	p-values
Age group			
18-30			
31-45	1	0.4-2.914	0.1
>45	0.21	0.06-0.7	0.01*
Workplace			
Housekeeping, Canteen & Maintenance			
Core Making	0.05	0.01-0.24	<0.01*
Melting & Moulding	0.24	0.05-1.29	0.01*
Polishing & Grinding	0.52	0.10- 2.81	0.45
Years of experience			
<1	1.37		
1-5	0.4	0.464.01	0.6
>5	0.88	0.11- 1.42	0.16
Self-reported personal protective equipment (PPE) use^	50.8	18.2-141.7	<0.01*

Binary logistic regression was performed on all variables significant on chi-square analysis (Table 4), with freedom from OSD as the modelled outcome. Workers older than 45 years were at higher risk than those aged 18-30 (adjusted odds ratio or AOR 0.21, 95% CI 0.06-0.7, p=0.01), whereas the 31-45 group did not differ significantly. By work section, core-making (AOR 0.05, 95% CI 0.01-0.24, p<0.01) and melting and moulding workers (AOR 0.24, 95% CI 0.05-1.29, p=0.01) were significantly less likely to remain disease-free than housekeeping, canteen and maintenance workers, while polishing and grinding workers did not differ significantly (AOR 0.52, p=0.45). Years of experience did not retain significance. In contrast, workers who reported using PPE were 50.8 times more likely to be free of OSD than non-users (AOR 50.8, 95% CI 18.2-141.7, p<0.01), making PPE use the strongest protective factor.

## Discussion

In this study of OSD and workplace risk of an iron-casting industry workforce, more than one in three workers (121/345, 35.1%) carried at least one clinically diagnosed OSD, with contact dermatitis being the single most common condition (15.1%) and prickly heat a close second (14.5%). The burden varied markedly by work section, peaking among core-making workers (65%). Self-reported use of PPE emerged as the strongest modifiable correlate of remaining disease-free (AOR 50.8, 95% CI 18.2-141.7; p<0.01).

The predominance of contact dermatitis is consistent with the wider literature, in which contact dermatitis constitutes the majority of all OSDs [3], with irritant or allergic contact dermatitis estimated to account for 70-95% of cases in Korean workers and for 70-90% in other occupational series [3,10]. Metalworking is an established high-risk occupation for irritant contact dermatitis, with oils, metalworking fluids, resins, and metal salts acting as cutaneous irritants and sensitizers [4]. The overall prevalence observed in our study is substantially higher than that derived from population-based self-report surveys. In the nationwide Korean working conditions survey, OSD affected roughly 1.35% of all workers and about 2.0% within manufacturing [10]. This gap most plausibly reflects two features of the present study: a high-exposure metal casting industry setting and case ascertainment through direct dermatological examination rather than self-report. The figure is, however, broadly comparable to the 27.5% prevalence found among informal Indonesian batik workers, in whom occupational contact dermatitis was likewise the commonest diagnosis [11], and lower than the 44.3% prevalence of itching reported among steel and power workers in Odisha [2], a difference attributable to the latter's symptom-based rather than clinician-confirmed outcome. A pooled European metalworker cohort, using a stringent definition of persistent rash, recorded skin symptoms in 10-13% of workers [4], in keeping with the contact dermatitis prevalence found here.

Prickly heat (miliaria), as the second most frequent OSD, reflects the thermally demanding nature of casting work. High ambient temperature and sweating are well-recognised predisposing factors for OSD [3], and high-temperature exposure was independently associated with OSD in Korean workers (OR 2.906) [10]. The salience of heat in this group is underscored by the Odisha data, in which heat stroke affected 48.3% of workers and 80.6% reported contact with hot metal [2], while miliaria was also documented among heat-exposed batik workers [11].

Burn scars (3.5%) are an expected consequence of contact with molten metal and hot surfaces, consistent with the burn injuries and pervasive hot-metal exposure described among steel workers [2]. Tinea (4.6%) is plausibly facilitated by sustained heat, humidity, and occlusive clothing, fungal infections being recognised among the infectious OSD [3]; in Asian settings in particular, tropical heat and humidity favor fungal work-related skin diseases [12]. Urticaria (2.6%), the least frequent condition, is compatible with contact urticarial reactions to workplace chemicals, also described in occupational dermatology [3].

The concentration of disease among core-making workers is biologically coherent, since core production involves intensive handling of chemical binders, resins, and sand-coating agents. Exposure to oil-based metalworking fluids and organic solvents has shown a clear exposure-response relationship with skin symptoms in metalworkers (prevalence ratios 1.76 and 2.06 for frequent use, respectively) [4], and skin contact with chemical products was independently associated with OSD in a Korean survey (OR 2.326) [10].

The association between self-reported PPE use and freedom from OSD was the most striking finding. Its direction is concordant with prior evidence, in which inadequate PPE use contributes to OSD risk [3] and work requiring protective equipment (a marker of hazardous exposure) was independently associated with OSD in Korean workers (OR 2.187) [10]. The very large effect seen here (AOR 50.8) should still be read with caution. Even though a real association likely exists, several things suggest this number overstates it: the confidence interval is very wide, PPE status was recorded only once (on a single day), and overall PPE use probably reflects a broader tendency toward safe behavior rather than the effect of PPE alone. We also cannot rule out reverse causation, that having skin disease influenced PPE use, rather than the other way around. Notably, no individual PPE item showed a significant association with OSD on observed single-item analysis; this dissociation accords with reports that occlusive items such as gloves can themselves provoke dermatitis [5] and that, among preventive measures, moisturisers and barrier creams have demonstrable protective effects whereas evidence for skin-protection education alone remains insufficient [13]. Consistent, comprehensive protection therefore appears to matter more than any single barrier. Previous studies have shown engineering controls targeting both heat (ventilation and cooling) and chemical exposure (enclosure and local exhaust), and skin-protection measures such as moisturisers and barrier creams are of proven preventive value [13]. However, the current study has not looked into these aspects.

OSD was somewhat more frequent in women than men (46.5% vs 33.4%), mirroring the Korean finding of higher OSD among female workers, however our findings were not statistically significant [10]. Conversely, male gender was identified as a risk factor for occupational contact dermatitis among batik workers, where men undertook the higher-exposure wet processes [14], underscoring that such associations are confounded by task allocation. Studies of Indian tannery workers have similarly attributed skin complaints chiefly to occupational exposure rather than to demographic factors [15]. OSD prevalence also rose with age, being highest in workers above 45 years (44%; p=0.027) and lowest in those with under one year of service (16%), a pattern compatible with cumulative cutaneous exposure tempered by a healthy-worker survivor effect, whereas age was not significant in the Korean cohort [10].

Strengths

The principal strength is diagnosis by a qualified dermatologist through a detailed history taking and individual clinical examination, providing more valid and complete case ascertainment than the self-reported or questionnaire-based approaches of comparable studies [2,10,16]. A high participation rate (83.3% of the entire registered workforce) and the census design minimise selection bias, while stratification by work section permits hazard-specific interpretation.

Limitations

The cross-sectional design precludes causal inference, and critically, allowed PPE use to be assessed only as same-day status rather than consistent adherence, which prospective designs capture far better [17]. Reliance on self-reported PPE use introduces social desirability and recall bias and raises the possibility of reverse causation. The study was conducted at a single metal casting factory, and the workforce was predominantly of middle SES (Class II) [9], limiting generalizability. Wide confidence intervals around several adjusted estimates indicate imprecision. And the absence of patch testing prevented differentiation of irritant from allergic contact dermatitis or identification of specific allergens such as the chromate frequently implicated in Indian occupational series [12].

## Conclusions

OSD affected more than a third of this metal casting industry workforce, dominated by contact dermatitis and concentrated among core-making workers, with PPE use being the leading modifiable protective factor. These findings argue for consistent, enforced, and section-appropriate PPE provision - particularly in core-making and melting areas - coupled with periodic dermatological screening. Future research should prioritize longitudinal or cohort designs with objective measurement of PPE adherence and patch testing to characterize causative allergens, alongside multi-center studies to establish the generalizability of these associations across the metal casting industry sector. A multi-center, longitudinal study or observations at different points of time, including all the possible different variables of the metal casting industry, would definitely provide more insights into OSDs in the metal casting industry. 

## Figures and Tables

**Figure 4:**
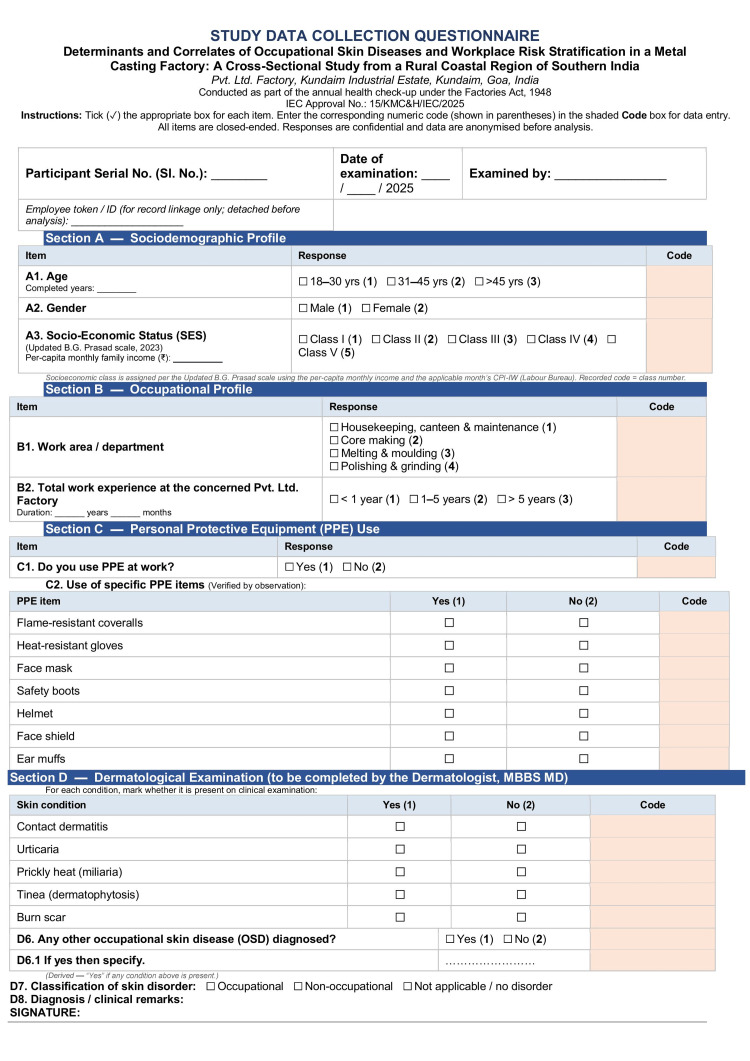
OSD Study Questionnaire
